# Ezetimibe Attenuates Atherosclerosis Associated with Lipid Reduction and Inflammation Inhibition

**DOI:** 10.1371/journal.pone.0142430

**Published:** 2015-11-10

**Authors:** Chunmiao Tie, Kanglu Gao, Na Zhang, Songzhao Zhang, Jiali Shen, Xiaojie Xie, Jian-an Wang

**Affiliations:** 1 Department of Cardiology, Cardiovascular Key Laboratory of Zhejiang Province, Second Affiliated Hospital, Zhejiang University School of Medicine, Hangzhou, Zhejiang, P.R. China; 2 Department of Clinical Laboratory, Second Affiliated Hospital, Zhejiang University School of Medicine, Hangzhou, Zhejiang, P.R. China; 3 Department of Cardiology, Affiliated Boai Hospital of Shaoxing University, Shaoxing, Zhejiang, P.R. China; Katholieke Universiteit Leuven, BELGIUM

## Abstract

**Background:**

Ezetimibe, as a cholesterol absorption inhibitor, has been shown protecting against atherosclerosis when combined with statin. However, side by side comparison has not been made to evaluate the beneficial effects of ezetimibe alone versus statin. Herein, the study aimed to test whether ezetimibe alone would exhibit similar effects as statin and the combination therapy would be necessary in a moderate lesion size.

**Methods and Results:**

ApoE-/- male mice that were fed a saturated-fat supplemented diet were randomly assigned to different therapeutic regimens: vehicle, ezetimibe alone (10 mg/kg/day), atorvastatin (20 mg/kg/day) or combination of ezetimibe and atorvastatin through the drinking water. On 28 days, mice were sacrificed and aorta and sera were collected to analyze the atherosclerotic lesion and blood lipid and cholesterol levels. As a result, ezetimibe alone exerted similar protective effects on atherosclerotic lesion sizes as atorvastatin, which was mediated by lowering serum cholesterol concentrations, inhibiting macrophage accumulation in the lesions and reducing circulatory inflammatory cytokines, such as monocyte chemoattractant protein (MCP-1) and tumor necrosis factor (TNF-α). In contrast to ezetimibe administration, atorvastatin alone attenuated atherosclerotic lesion which is dependent on its anti-inflammation effects. There were no significance differences in lesion areas and serum concentrations of cholesterol, oxidized LDL and inflammatory cytokines between combination therapy and monotherapy (either ezetimibe or atorvastatin). There were significant correlations between the lesion areas and serum concentrations of cholesterol, MCP-1 and TNF-α, respectively. However, there were no significant correlations between the lesion areas and serum concentrations of TGF-β1 and oxLDL.

**Conclusions:**

Ezetimibe alone played the same protection against a moderate atherosclerotic lesion as atorvastatin, which was associated with lowering serum cholesterol, decreasing circulating inflammatory cytokines, and inhibiting macrophage accumulation in the lesions.

## Introduction

Ezetimibe, a cholesterol absorption inhibitor that is widely used in treating patients with family hypercholesterolemia, targets a polytopic transmembrane protein localized at the apical membrane of enterocytes and the canalicular membrane of hepatocytes, called Niemann-Pick C1-Like 1 (NPC1L1) protein [1]. Since NPC1L1 protein functions as a sterol transporter to mediate intestinal cholesterol absorption and counter-balances hepatobiliary cholesterol excretion, ezetimibe exerts the dual effects on lipid metabolism, inhibiting exogenous cholesterol absorption from diet while promoting a compensatory increase of endogenous cholesterol synthesis, the net effects of these two process result in a reduction of low density lipoprotein (LDL) as well as total cholesterol concentrations in humans [2]. Interestingly, addition of ezetimibe to statin treatment can further reduce LDL and cholesterol concentrations and achieve the target values satisfactorily in patients with atherosclerotic cardiovascular disease (ASCVD) [3].

Ezetimibe has been thoroughly investigated in clinical trials to evaluate its effects on lipid profiles [3–4], cardiovascular outcomes [5] and/or other surrogate endpoints [6–7]. In fact, in most of these trials, ezetimibe has been added to statin as a combination therapy. “SHARP” study has shown that combination of ezetimibe and statin resulted in a 17% reduction in major atherosclerotic events in patients with chronic kidney disease [6]. However, some of the clinical studies, such as ENHANCE [4], SANDS [7] and VYCTOR [8] trials demonstrated that this combination therapy failed to show beneficial effect on carotid intima-media thickness. In contrast, IMPROVE-IT trial showed that the addition of ezetimibe versus placebo to simvastatin therapy had a 6.4% lower risk of major cardiovascular events in patients with acute coronary syndromes (ACS), including subsequent heart attack, stroke, cardiovascular death, re-hospitalization for unstable angina and procedures to restore blood flow to the heart [5].

Up to now, it remains unclear whether "high-intensity cholesterol-lowering therapy", i.e. ezetimibe, a lipid-lowering drug, combined statin which aims to intensify the cholesterol lowering effect could result in a greater effects compared with the "high-intensity statin therapy", i.e. an intensified statin therapy. Studies have already reported that addition of ezetimibe to statin administration facilitates the reduction in LDL cholesterol concentrations [3], which also contributes to significantly improve cardiovascular outcomes in the ACS patients [5]. However, side by side comparison has not been made to evaluate the beneficial effects of the ezetimibe monotherapy versus statin in the pathogenesis of atherosclerotic diseases. Moreover, the synergistic effects of ezetimibe and statin have already been demonstrated in either an apoE or a LDL receptor knock-out mouse model [9]. The effects were tested when mice were fed a fat-enriched diet for as long as 6 months [10]. Based on the above information, therefore, in the present study, we sought to test whether: 1) ezetimibe alone would exhibit similar effects as statin. An apoE-/- mice was specifically used to investigate whether differential effects exist between ezetimibe and atorvastatin when the mice were fed on a fat-enriched diet; 2) the combination of ezetimibe and atorvastatin therapy would be necessary when these apoE mice were fed on the high fat diet for a short period which results in a moderate atherosclerotic lesion size.

## Materials and Methods

### Mice and diet

Male apoE-/- mice on C57BL/6 background were purchased from the Beijing Vital River Laboratory Animal Technology Corporation and bred in-house. Mice were housed under specific pathogen-free (SPF) conditions, and fed a normal laboratory diet. One week prior to drug administration, all mice started a diet containing milk fat (21% wt/wt) and cholesterol (0.15% wt/wt, Beijing Keaoxieli Corp., Beijing, China), which maintained for 5 weeks.

### Study design

Forty-eight male apoE-/- mice (8–12 weeks old) were randomly divided into four groups as follows: 1) ezetimibe group (n = 12, kindly provided by Merck Sharp & Dohme Pty Ltd, China); 2) atorvastatin group (n = 12, kindly provided by Pfizer Biopharmaceutical Co., China); 3) combination of ezetimibe and atorvastatin (n = 12, combination therapy) and 4) control group mice that received placebo therapy (n = 12). All the pharmacological intervention started 7 days after initiation of the fat-enriched diet feeding and continued until the end of the whole study. Mice were inspected daily and weighed weekly. Ezetimibe and atorvastatin were dissolved in drinking water at concentrations that gave approximate doses of 10 mg/kg/day and 20 mg/kg/day, respectively, delivered by daily gavage [11–12]. Mice were terminated after 28 days of drug administration, being anesthetized by intraperitoneal injection of pentobarbital sodium (60 mg/kg).

### Blood pressure monitoring

Systolic blood pressures were measured on conscious mice using noninvasive tail-cuff systems (CODA 6; Kent Scientific Corp, Torrington, CT, U.S.A.) as described previously [11, 13–14]. Systolic blood pressures were measured one week before drug administration to record baseline blood pressures, and repeated on weeks 4 during drug administrations.

### Serum measurements

Serum cholesterol concentrations were determined using an enzymatic assay kit (TC, catalog number OSR6216E, Beckman Coulter Inc. California, U.S.A) as describe previously [11–12]. Serum concentrations of alanine aminotransferase were measured by a lactic acid dehydrogenase enzymatic method (ALT, catalog number OSR6107E, Beckman Coulter, Inc. California, U.S.A). Serum concentrations of aspartate transaminase were measured by malate dehydrogenase enzymatic method (AST, catalog number OSR6209E, Beckman Coulter, Inc. California, U.S.A). Serum concentrations of monocyte chemoattractant protein-1 (MCP-1, catalog number MJE00, R&D Systems, Minnesota, U.S.A), tumor necrosis factor (TNF-α, catalog number MTA00B, R&D Systems, Minnesota, U.S.A) and transforming growth factor (TGF-β1, catalog number MB100B, R&D Systems, Minnesota, U.S.A) were measured with ELISA kits according to manufacturer’s recommendation, respectively [11–12]. Serum oxidized LDL concentrations were measured with an ELISA kit according to manufacturer’s recommendation (oxLDL, catalog number E90527Mu, Cloud-Clone Corp. Texas, U.S.A).

### Atherosclerosis analysis

Mice were terminated after 28 days of drug administration, with blood harvested from left ventricles and aortic arches fixed with10% neutral buffer formalin. Aortic roots were embedded in O.C.T. and frozen at -20°C. Atherosclerosis was assessed using two methods: (1) percent lesion areas in the ascending and aortic arch and part of descending aorta using en face technique and (2) also lesion areas of the aortic root using serial cross-sections as described previously [11, 13–14]. Oil Red O staining was used to assist in visualization of lesions. Quantitative analysis of atherosclerosis was performed using Image-Pro software (Media Cybernetics, Bethesda, MD, U.S.A) as described previously [11, 13–14]. Macrophages in atherosclerotic plaques on aortic root were detected by immunostaining with rabbit anti-CD68 (catalog number ab12512, Abcam, Cambridge, MA, U.S.A.) as described previously [11–12]. Immunostaining was performed with a commercially available system (Fisher Microprobe, Pittsburgh, PA, U.S.A). A peroxidase-based ABC system and the brown chromogen, DAB, were used to visualize the antigen-antibody reaction. Macrophage content in atherosclerotic lesions was graded as follows: 0 = no staining, 1 = slight staining, 2 = mild staining, 3 = moderate staining and 4 = abundant staining (S5 Fig).

### Statistical analyses

Data are presented as means ± standard error of means (SEM). SigmaPlot version 12.5 (Systat Software Inc., San Jose, CA, USA) was used for statistical analyses. Data were tested for use of parametric or non-parametric post hoc analysis and then analyzed by One-way ANOVA. The correlations between the atherosclerotic lesion areas and serum concentrations of cholesterol, inflammatory cytokines were examined by two-tailed Pearson Correlation, respectively. A P< 0.05 was considered to be statistically significant.

### Ethics statement

All mouse studies were performed with approval of Zhejiang University Institutional Animal Care and Use Committee.

## Results

### Atherosclerotic lesion characteristics

**Fig 1:**
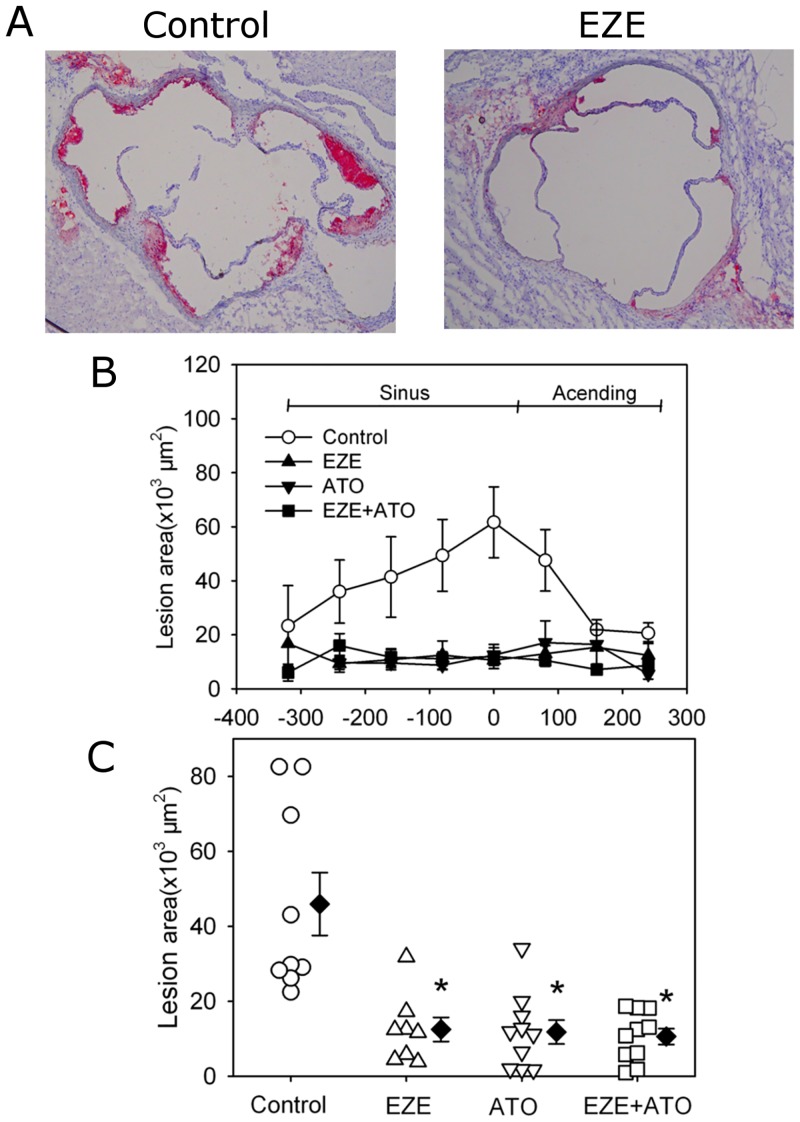
Drugs attenuated atherosclerotic lesion areas of aortic roots. Atherosclerosis was assessed using cross-sections (10 μm thick) of aortic roots from aortic sinuses to ascending aorta. (A) Representative tissue sections by Red Oil O staining used to assist in visualization of lesions in aortic roots. Left panel indicated the mice in control group (CTL), whereas right panel indicated the mice in ezetimibe group (EZE). (B) The distributions of atherosclerotic lesions from the aortic sinuses to ascending aorta between four groups. (C) Average lesion areas in the mice of four groups. Circles represent the values of individual mice, diamonds represent means, and bars are SEM. N = 9, 8, 11 and 11 for vehicle (Control), ezetimibe (EZE), atorvastatin (ATO), and combination (EZE+ATO) groups, respectively. Quantitative analysis of atherosclerosis was performed using Image-Pro software. Statistical analysis was performed using one way repeated measure ANOVA. * *P*<0.001, compared with control group.

**Fig 2:**
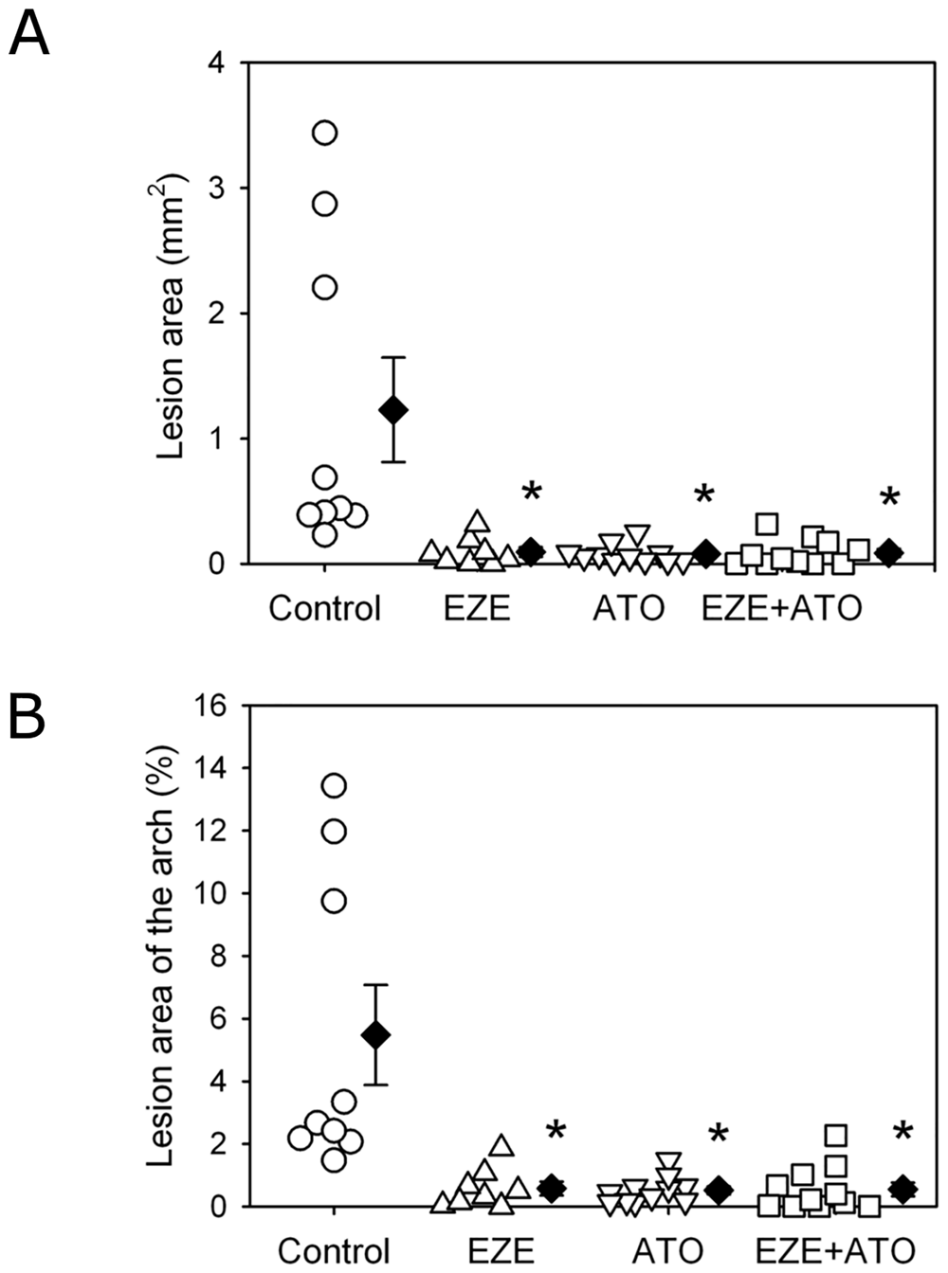
Drugs attenuated atherosclerotic lesion areas in aortic arches. Atherosclerosis was assessed on aortic arches by en face technique, including lesion area and percent lesion areas on intimas of the ascending, aortic arch region and part of descending aorta. (A) Lesion areas in the mice between four groups; (B) Percent lesion area of the aortic arch area in the mice between four groups. Circles represent the values of individual mice, diamonds represent means, and bars are SEM. N = 9, 8, 11 and 11 for vehicle (Control), ezetimibe (EZE), atorvastatin (ATO), and combination (EZE+ATO) groups, respectively. Quantitative analysis of atherosclerosis was performed using Image-Pro software. Statistical analysis was performed using one way repeated measure ANOVA. * *P*<0.001, compared with control group.

Forty-eight male apoE -/- mice were fed the fat-enriched diet and administered the vehicle water, ezetimibe (10 mg/kg/day), atorvastatin (20 mg/kg/day), and combination therapy, respectively. During the 28 days of drug administration, there was no statistical significance of mortalities between the four groups (P>0.05 by Fisher Exact Test). Neither body weight nor systolic blood pressure was affected by drugs administration (S1 and S2 Figs). As expected, one month fat-enriched diet accelerated the formation of atherosclerotic lesions in apoE -/- mice for a short period study, as shown in aortic roots (from aortic sinuses to ascending aorta) and percent lesion areas of aortic arches (Figs 1 and 2). Compared to the atherosclerotic lesion areas in apoE-/- mice with angiotensin II infusion for 28 days in our published study previously [11–12], the atherosclerotic lesion areas were mild to moderate in our present study, which could mimick a natural pathological process in clinical patients, thus the data obtained with this model would be more of clinical applications.

### Reduction on atherosclerotic lesions

Atherosclerosis was evaluated by the lesion areas in aortic roots (from aortic sinuses to ascending aorta) and percent lesion areas of aortic arches. Compared to those of the mice administered with vehicle water (control group), the atherosclerotic lesion areas in aortic roots were significantly decreased in mice administered with ezetimibe, atorvastatin or combination therapy, respectively (Fig 1). However, there was no significant difference in lesion areas between any two groups among the three different therapies. When the percent lesion area of aortic arches was assessed, the same pattern of changes was observed as the lesion size in aortic roots (Fig 2 and S3 Fig). Thus, our data suggested that in the setting of mild to moderate size of atherosclerotic lesions, the combination therapy did not show any extra benefits compared with drug monotherapy.

### Differential effects on serum cholesterol

**Fig 3:**
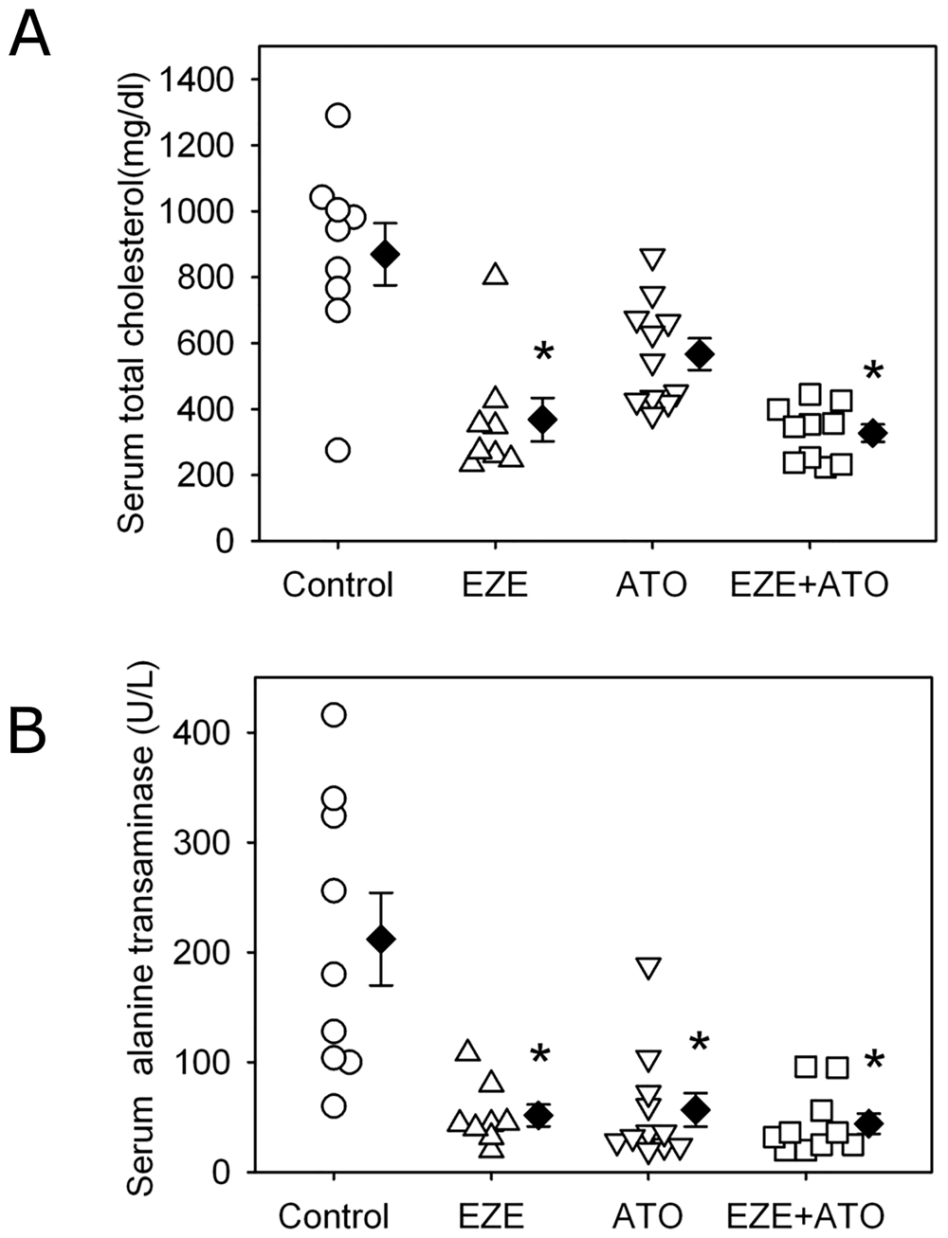
Differential effects on serum concentrations of cholesterol and hepatic enzyme. (A) Serum total cholesterol concentrations were measured using an enzymatic assay kit according to manufacturer’s recommendation. (B) Serum concentrations of alanine aminotransferase were measured by a lactic acid dehydrogenase enzymatic method. Circles represent the values of individual mice, diamonds represent means, and bars are SEM. N = 9, 8, 11 and 10 for vehicle (Control), ezetimibe (EZE), atorvastatin (ATO), and combination (EZE+ATO) groups, respectively. Statistical analysis was performed using one way repeated measure ANOVA. * *P*<0.001, compared with control group.

Serum cholesterol concentrations were determined to compare the pharmacological effects of both drugs and explain the potential mechanisms of anti-atherosclerotic effects. Compared to the mice administered with vehicle water, both ezetimibe alone and combination therapy significantly reduced serum concentrations of total cholesterol (Fig 3). There was no significant difference of serum cholesterol concentrations between the mice administered with ezetimibe alone and combination of ezetimibe and atorvastatin. Consistent with the previous reports and our own published data [11,15], administration of atorvastatin alone did not significantly reduce serum concentrations of total cholesterol in these apoE-/- mice, compared to vehicle.

Serum enzymes were also measured for the four groups, including alanine aminotransferase and aspartate transaminase in order to rule out potential drug-induced hepatic damage. Both ezetimibe alone and combination therapy actually resulted in a decrease of serum alanine aminotransferase concentrations, compared to vehicle (Fig 3). Serum concentrations of aspartate transaminase were not affected by either ezetimibe or atorvastatin alone. However, compared to vehicle, combination therapy significant reduced serum concentrations of aspartate transaminase (S4 Fig).

### Inflammatory cytokines and macrophage accumulation in the lesions

**Fig 4:**
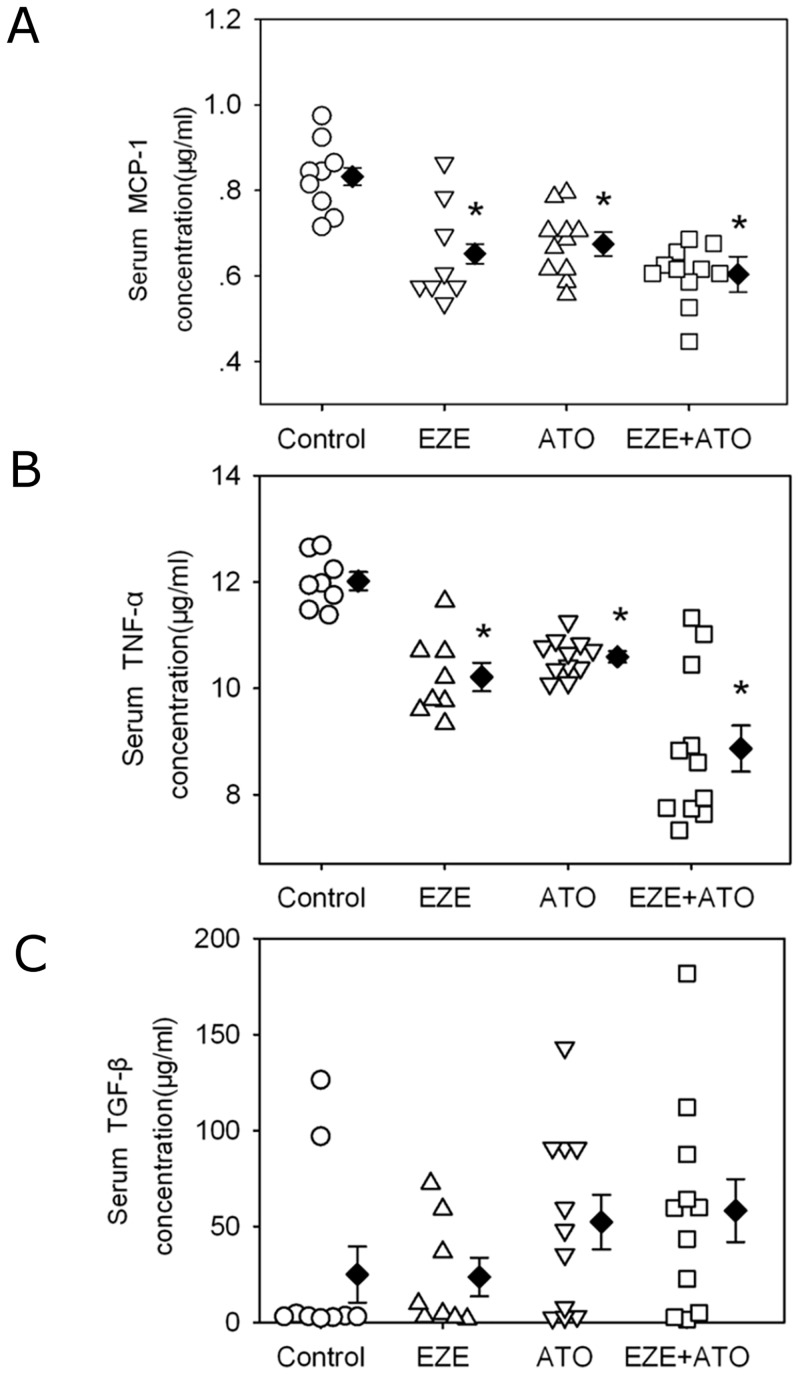
Drugs reduced serum inflammatory cytokines. Serum concentrations of inflammatory cytokines were measured using ELISA kits, including (A) monocyte chemoattractant protein-1 (MCP-1), (B) tumor necrosis factor (TNF-α) and (C) transforming growth factor (TGF-β1). Circles represent the values of individual mice, diamonds represent means, and bars are SEM. N = 9, 8, 11 and 11 for vehicle (Control), ezetimibe (EZE), atorvastatin (ATO), and combination (EZE+ATO) groups, respectively. Statistical analysis was performed using one way repeated measure ANOVA. * *P*<0.001, compared with control group.

To explore the potential mechanisms of these drugs on attenuating atherosclerosis, serum concentrations of MCP-1, TNF-α and TGF-β1 were determined. Consistently, serum concentrations of MCP-1and TNF-α were reduced respectively by administration of ezetimibe, atorvastatin or combination therapy, compared to vehicle (Fig 4). However, there were no significant differences of serum MCP-1and TNF-α concentrations in the mice administered with ezetimibe, atorvastatin or combination therapy. Neither ezetimibe nor atorvastatin administration affected serum TGF-β1concentrations.

**Fig 5:**
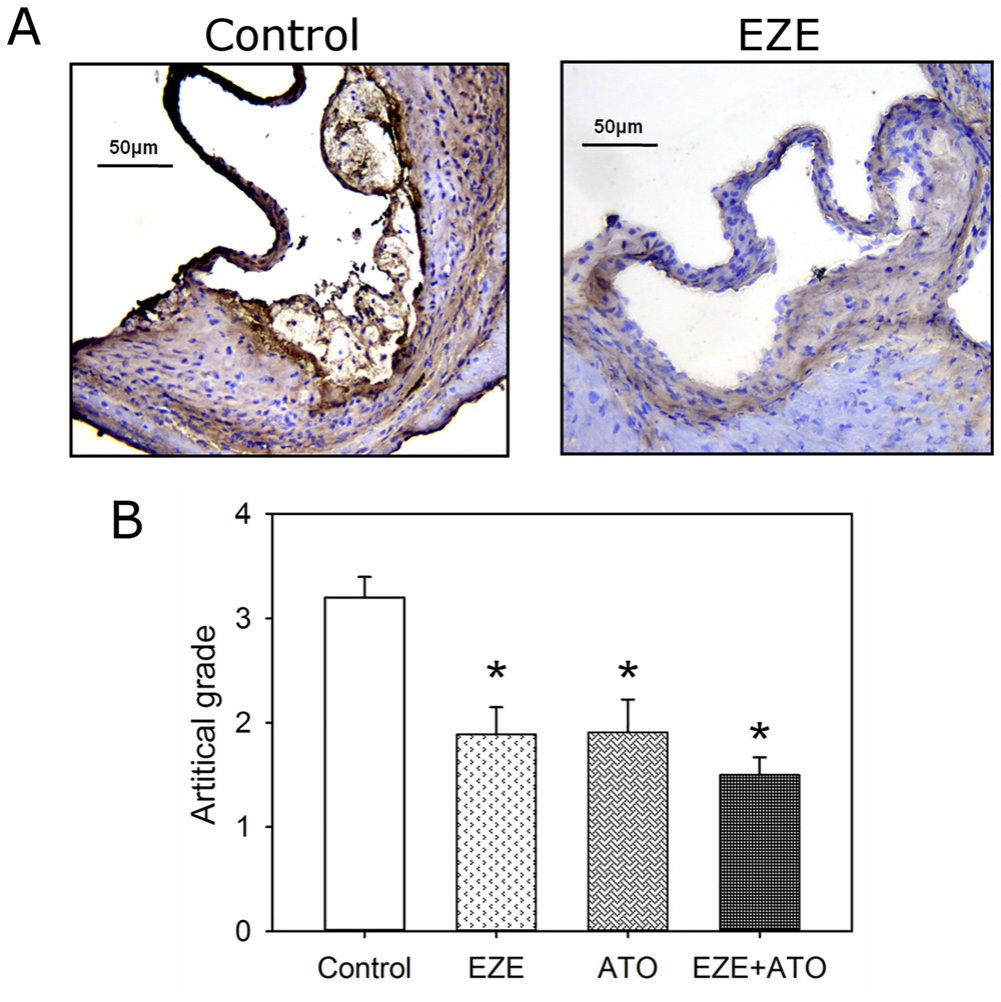
Drugs suppressed macrophage accumulation in atherosclerotic lesions. Macrophage (CD68) was detected by immunostaining on cross-sections (10 μm thick) of aortic roots. According to artificial grades of positive immunostaining (as shown in S5 Fig), macrophage contents were assessed semiquantitatively. (A) Representative tissue sections by immunostaining. Left panel indicated the mice in control group, whereas right panel indicated the mice in ezetimibe group (EZE). (B) Artificial grades of macrophage contents in lesions of the mice. Histobars represent means, and error bars represent SEM. N = 9, 8, 11 and 11 for vehicle (Control), ezetimibe (EZE), atorvastatin (ATO), and combination (EZE+ATO) groups, respectively. Statistical analysis was performed using one way repeated measure ANOVA. * *P*<0.001, compared with vehicle.

Consistent with our previous report [11], atherosclerotic lesions in apoE-/- mice on fat-enriched diet have macrophages presented. The accumulation of macrophages in the lesions was significant attenuated in the mice administered with ezetimibe, atorvastatin or combination therapy, compared to those administered with vehicle (Fig 5 and S5 Fig). However, there was no significant difference of macrophage accumulation in lesions between the three different therapeutic regimens.

### Correlation of lesion areas with serum concentrations of cholesterol and inflammatory cytokines

**Fig 6:**
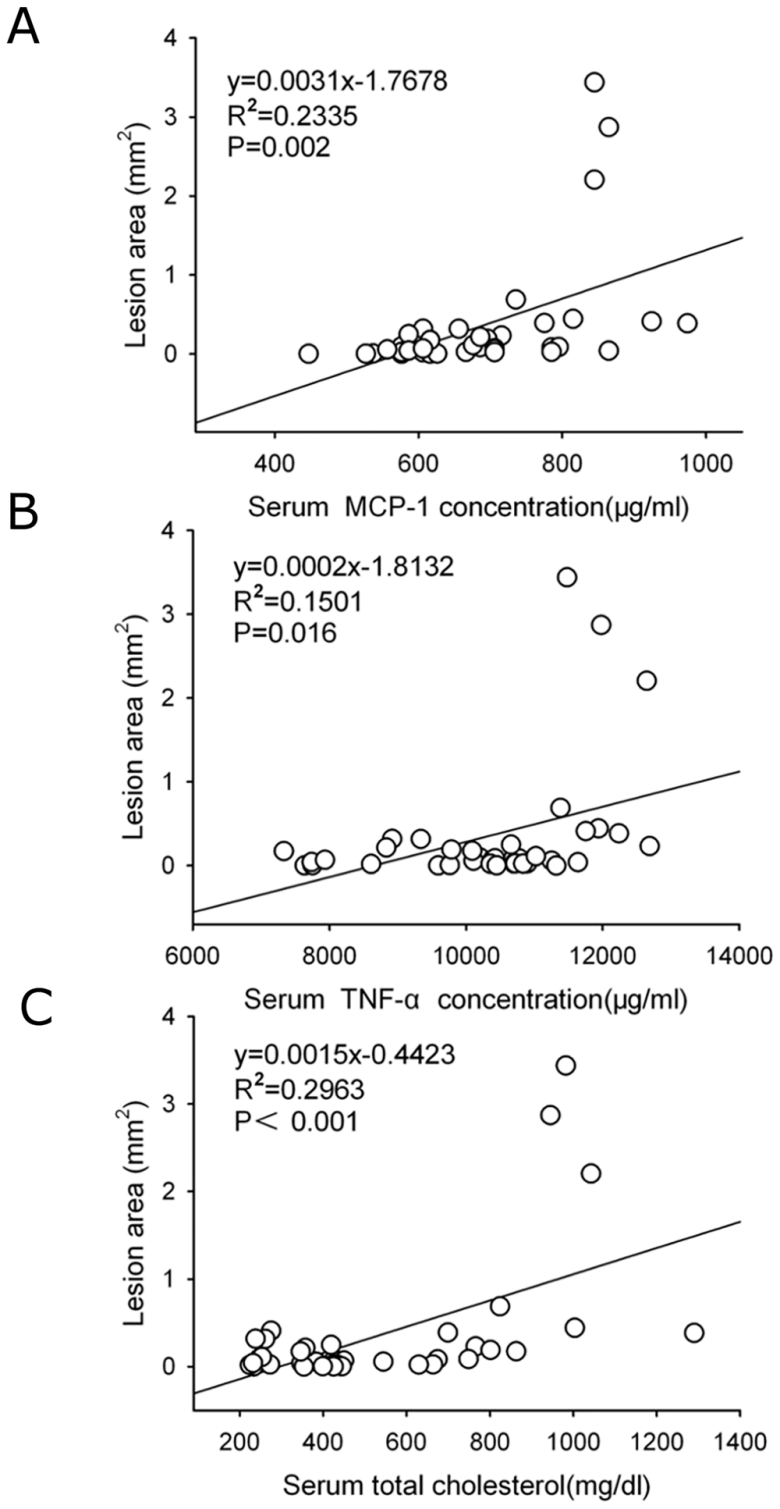
Correlation of atherosclerotic lesion areas with serum concentrations of cholesterol and inflammatory cytokines. The correlations between the atherosclerotic lesion areas and (A) serum concentrations of cholesterol, inflammatory cytokines including (B) monocyte chemoattractant protein-1 (MCP-1), and (C) tumor necrosis factor (TNF-α), respectively. Statistical analysis was performed using two-tailed Pearson correlation. R indicated correlation coefficient.

The pathogenesis factors involved in atherosclerosis have been identified, such as hyperlipidemia, oxidation of LDL and inflammation [16]. Serum oxLDL concentrations were also determined to explore the potential mechanisms of protection against atherosclerosis by these two drugs. Interestingly, compared to those of vehicle, serum oxLDL concentrations were not significantly different in the mice administered with ezetimibe, atorvastatin or combination of both drugs (S6 Fig). There were significant correlations between the atherosclerotic lesion areas and serum cholesterol concentrations (r = 0.546, P< 0.001), serum MCP-1 concentrations (r = 0.483, P = 0.002) and serum TNF-α concentrations (r = 0.388, P = 0.016, Fig 6). There were no significant correlations between the atherosclerotic lesion areas and serum concentrations of TGF-β1 (P = 0.156) and oxLDL (P = 0.482).

## Discussion

As a commonly used cholesterol absorption inhibitor, ezetimibe can decrease serum cholesterol. In clinical practice, ezetimibe has often been used in combination with statins to treat ASCVD patients [4, 6–7,17]. On the other hand, a wealth body of evidence has shown that statin exerts its anti-atherosclerotic effects by lowering LDL-C concentrations, attenuating inflammation, and protecting endothelial function [18]. However, no studies have directly compared the effects of ezetimibe alone on atherosclerotic lesions with statin alone or combination therapy (i.e., ezetimibe combined with atorvastatin). In addition, no data was available to validate whether the combination therapy would be necessary to protect against a moderate lesion size. Thus, our study for the first time provided evidence that ezetimibe alone can achieve the similar effects as atorvastatin, yet with a moderate atherosclerotic lesion size, and show that no further benefits could be observed when a combination therapy was used. It is of note that our data showed that ezetimibe exhibited both lipid-lowering and anti-inflammatory effects, while atorvastatin mainly demonstrated its anti-inflammatory effects, even though both result in a reduction in atherosclerotic lesions.

### The differential effects between ezetimibe and atorvastatin

As a specific inhibitor of intestinal membrane NPC1L1 protein, ezetimibe is widely applied in primary hypercholesterolemic patients to control serum LDL concentrations. A growing body of evidence provides mechanistic insights that anti-atherosclerotic effects of ezetimibe may be mediated by lowering cholesterol concentrations, protecting endothelial function, and decreasing inflammatory parameters [19–21]. In the pathogenesis of atherosclerotic lesions, the roles of ox-LDL were shown to initiate endothelial dysfunction, recruit circulatory mononuclear cells, promote macrophage accumulation, and produce inflammatory cytokines with oxidative stress damage [22]. The present study demonstrated that administration of ezetimibe alone exerted the same effects of attenuating a moderate atherosclerotic lesion area as statin in hypercholesterolemic mice. Although ezetimibe alone did not decrease serum ox-LDL concentrations, which acts as a circulatory marker of oxidation stress reaction, both serum total cholesterol concentrations and circulating inflammation markers, such as MCP-1 and TNF-α, were significantly reduced by ezetimibe alone. Thus, our data indicate that ezetimibe protected against atherosclerosis by reducing cholesterol and controlling inflammation.

On the other hand, in this hypercholesterolemic mice model, atorvastatin did not affect serum total cholesterol concentrations, which is consistent with the previous reports [11,15]. Yet, atorvastatin significantly lowered inflammatory cytokines concentrations, while no effect on serum ox-LDL concentrations. Interestingly, the reduction in atherosclerotic lesion areas was similar between the three different therapeutic regimens. Thus, in the present study, atorvastatin attenuated atherosclerotic lesions mainly through its anti-inflammatory effects, independent of lipid-lowering and oxidation suppressing.

### Synergistic effects by combination therapy in moderate atherosclerotic lesions

It has been reported that ezetimibe 10 mg plus simvastatin 20 mg daily produced a 17% reduction in major atherosclerotic events in patients with chronic kidney disease during a median of 4.9 years’ follow-up [6]. However, the same dose of ezetimibe combined with simvastatin 40 mg daily did not change carotid intima-media thickness in high-risk coronary patients [8]. In the “IMPROVE-IT” trial, the role of ezetimibe was evaluated for ACS patients. It has showed that combination therapy had a 6.4% lower risk of major cardiovascular events in ACS patients, which was the first clinical trials showing beneficial improved outcome of for ezetimibe combined with statin [5]. Being consistent with the previous reports [7, 23–24], our present study showed that combination therapy did not exert synergistic effect on a moderate atherosclerotic lesion size, as well as serum concentrations of cholesterol, oxLDL and inflammation cytokines, implying that combination therapy might not be necessary for patient with a mild to moderate size of atherosclerotic lesion in clinical practice.

### Study limitations

Based on our hypothesis, the present study was designed to only look at a mice model with moderate atherosclerotic lesion size. Thus, all three therapeutic strategies exhibited benefits against atherosclerosis. Moreover, even single pharmacological interventions, i.e. ezetimibe monotherapy, and atorvastatin monotherapy respectively, showed similar degree of potency as the combination therapy. This might be a different story if more than eight weeks of fat-enriched diet was given to these apoE deficient mice. This actually has already been tested in previous studies with conflicting results [25]. Therefore, it should always be born in mind that the lesion severity should be considered when the potency or efficiency of different therapeutic regimens was chosen. Also, our present study failed to further look into the detailed molecular mechanisms due to limited abilities.

In conclusion, ezetimibe alone played the same protection against a moderate atherosclerotic lesion as atorvastatin. However, combination of ezetimibe with atorvastatin does not show synergistic effects on a moderate atherosclerosis. The anti-atherosclerotic effects of ezetimibe might be associated with lowering serum cholesterol, decreasing circulatory inflammation cytokines, and inhibiting macrophage accumulation in lesions.

## Supporting Information

S1 Fig Drugs had no effects on mice body weight gain. Mice were fed fat-enriched diet and weighed weekly through the whole study. Vehicle or drug administration in drinking water by daily gavage started after one week of fat-enriched diet. Circles represent mean values at each time point and bars represent SEM. N = 9, 8, 11 and 11 for vehicle (Control), ezetimibe (EZE), atorvastatin (ATO), and combination (EZE+ATO) groups, respectively. Statistical analysis was performed using one way repeated measure ANOVA. P >0.05 for the comparisons between the four groups.
(TIF)

S2 Fig Drugs did not alter systolic blood pressure. Systolic blood pressures were measured using a tail-cuff system prior to drug administration (week 0) and at week 4 during the study. Vehicle or drug administration in drinking water by daily gavage started after one week of fat-enriched diet. Histobars represent means, and error bars represent SEM. N = 9, 8, 11 and 11 for vehicle (Control), ezetimibe (EZE), atorvastatin (ATO), and combination (EZE+ATO) groups, respectively. Statistical analysis was performed using one way repeated measure ANOVA. P >0.05 for the comparisons between the four groups.
(TIF)

S3 Fig Representative images for the lesions in aortic arches. Atherosclerosis was assessed on aortic arches by en face technique, including lesion area and percent lesion areas on intimas of the ascending, aortic arch region and part of descending aorta. Representative tissue sections by Red Oil O staining used to assist in visualization of lesions in aortic arches. Upper panel indicated the mice in control group, whereas lower panel indicated the mice in ezetimibe group (EZE).
(TIF)

S4 Fig Combination therapy decreased serum concentrations of aspartate transaminase. Serum concentrations of aspartate transaminase were measured by malate dehydrogenase enzymatic method. Circles represent the values of individual mice, diamonds represent means, and bars are SEM. N = 9, 8, 11 and 10 for vehicle (Control), ezetimibe (EZE), atorvastatin (ATO), and combination (EZE+ATO) groups, respectively. Statistical analysis was performed using one way repeated measure ANOVA. ** P<0.05; compared with vehicle.
(TIF)

S5 Fig Classification of macrophage content in the lesions by immunostaining. In the lesions, macrophage (CD68) was detected by immunostaining on cross-sections (10 μm thick) of aortic roots. Macrophage content in atherosclerotic lesions was graded as follows: 0 = no staining, 1 = slight staining, 2 = mild staining, 3 = moderate staining and 4 = abundant staining. Artificial classification (grade 1–4) by immunostaining was used to evaluate macrophage contents in the lesions. CD68 positive staining as shown brown in the lesions represents macrophage.
(TIF)

S6 Fig Drugs did not reduce serum oxLDL concentrations. Serum oxLDL concentrations were measured using an ELISA kit. Circles represent the values of individual mice, diamonds represent means, and bars are SEM. N = 9, 8, 11 and 10 for vehicle (Control), ezetimibe (EZE), atorvastatin (ATO), and combination (EZE+ATO) groups, respectively. Statistical analysis was performed using one way repeated measure ANOVA.
(TIF)
