# Supplementary figures and images for: Ezetimibe Attenuates Atherosclerosis Associated with Lipid Reduction and Inflammation Inhibition
Source: PLoS One. 2015 Nov 10;10(11):e0142430. doi: 10.1371/journal.pone.0142430 (PMC4640821; doi:10.1371/journal.pone.0142430)

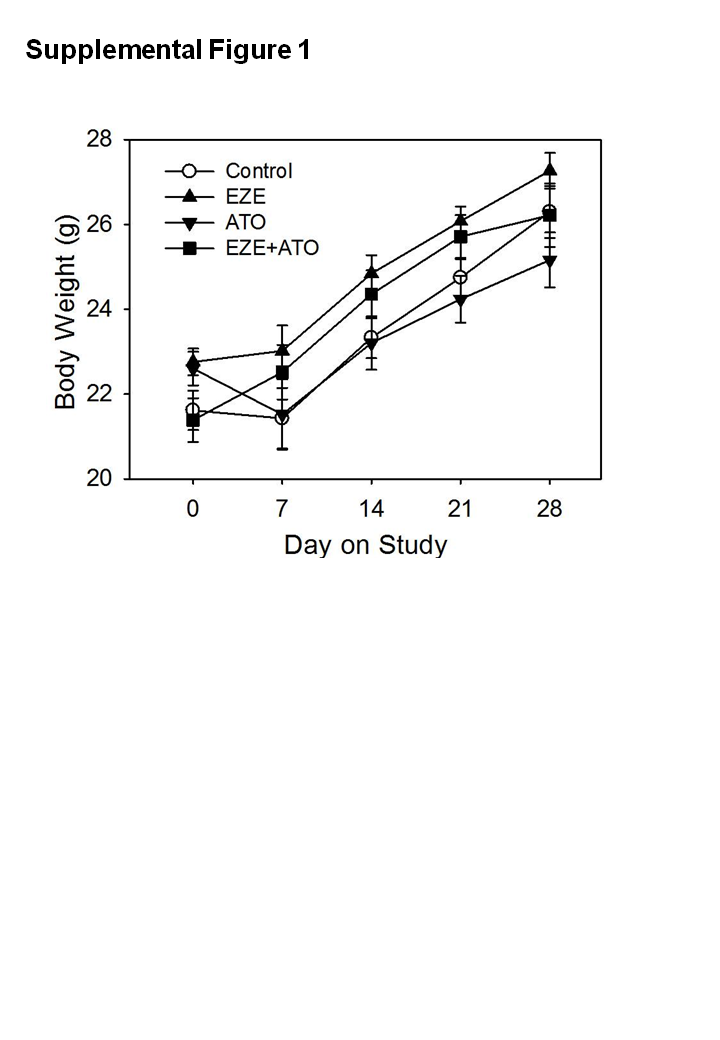

Supplement: S1 Fig — Mice were fed fat-enriched diet and weighed weekly through the whole study. Vehicle or drug administration in drinking water by daily gavage started after one week of fat-enriched diet. Circles represent mean values at each time point and bars represent SEM. N = 9, 8, 11 and 11 for vehicle (Control), ezetimibe (EZE), atorvastatin (ATO), and combination (EZE+ATO) groups, respectively. Statistical analysis was performed using one way repeated measure ANOVA. P >0.05 for the comparisons between the four groups. (TIF) [file pone.0142430.s001.TIF]

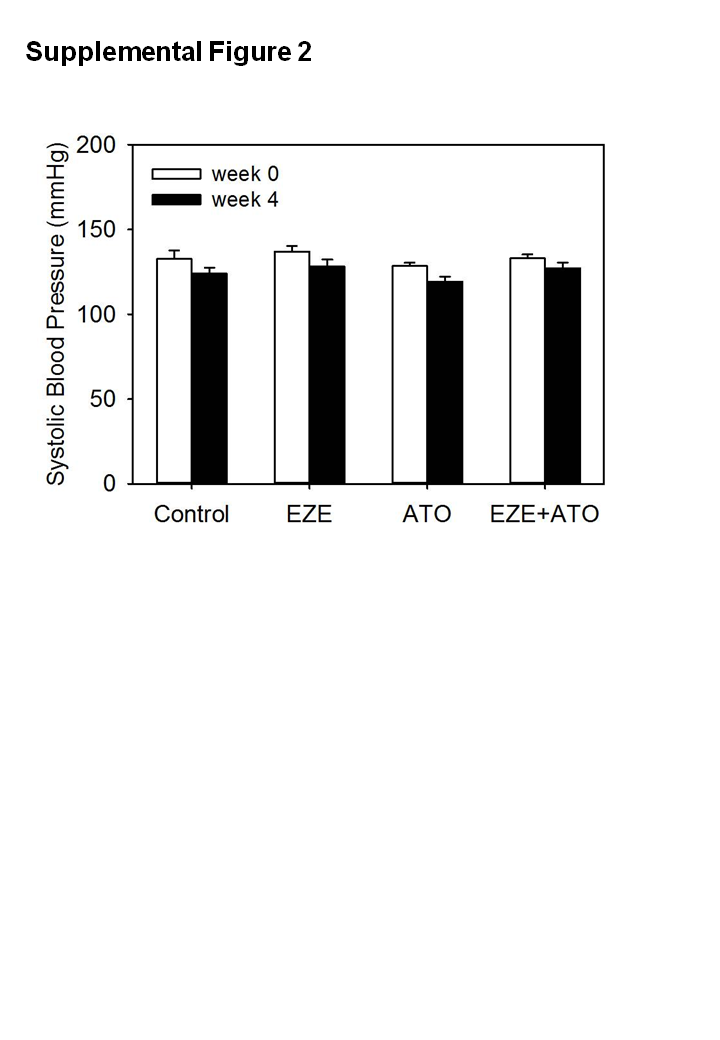

Supplement: S2 Fig — Systolic blood pressures were measured using a tail-cuff system prior to drug administration (week 0) and at week 4 during the study. Vehicle or drug administration in drinking water by daily gavage started after one week of fat-enriched diet. Histobars represent means, and error bars represent SEM. N = 9, 8, 11 and 11 for vehicle (Control), ezetimibe (EZE), atorvastatin (ATO), and combination (EZE+ATO) groups, respectively. Statistical analysis was performed using one way repeated measure ANOVA. P >0.05 for the comparisons between the four groups. (TIF) [file pone.0142430.s002.TIF]

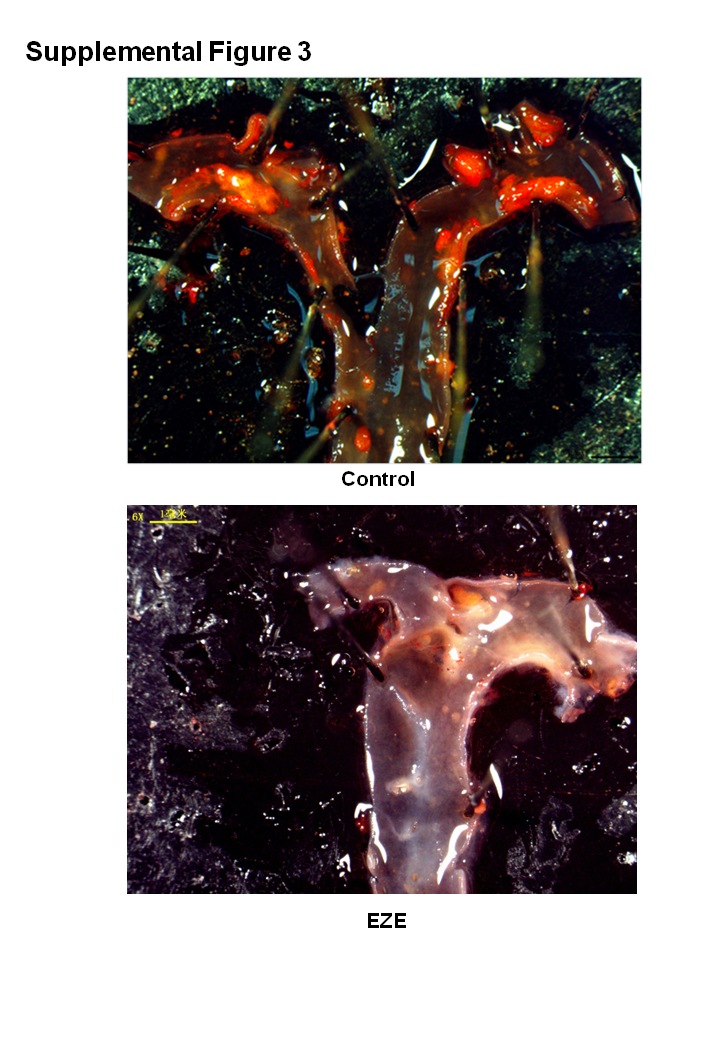

Supplement: S3 Fig — Atherosclerosis was assessed on aortic arches by en face technique, including lesion area and percent lesion areas on intimas of the ascending, aortic arch region and part of descending aorta. Representative tissue sections by Red Oil O staining used to assist in visualization of lesions in aortic arches. Upper panel indicated the mice in control group, whereas lower panel indicated the mice in ezetimibe group (EZE). (TIF) [file pone.0142430.s003.TIF]

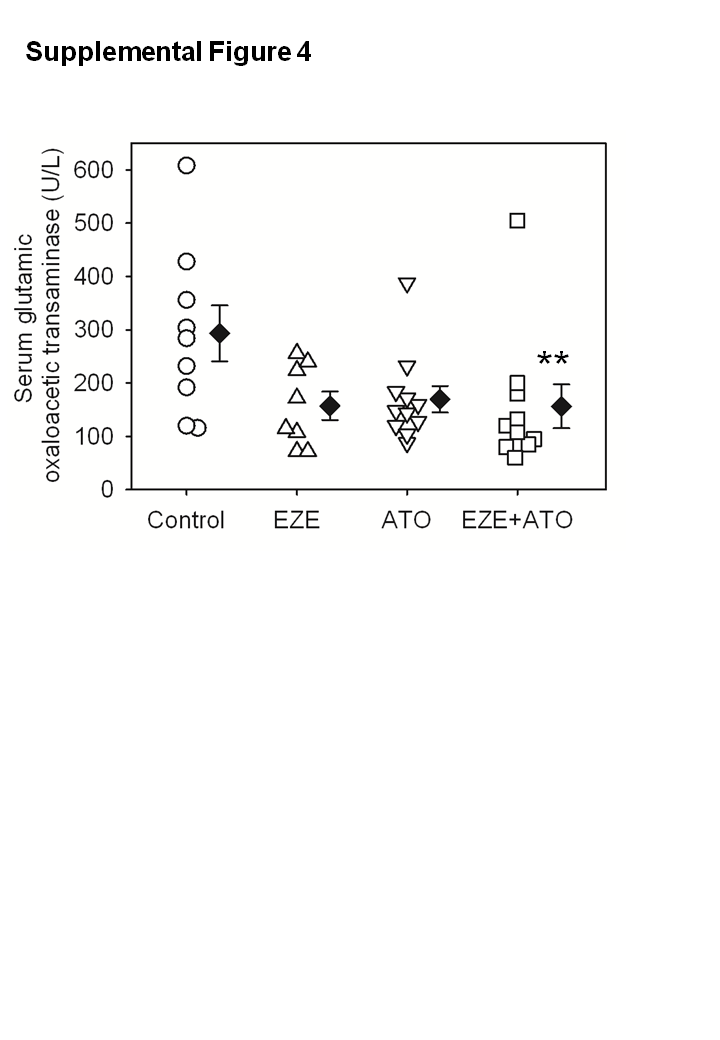

Supplement: S4 Fig — Serum concentrations of aspartate transaminase were measured by malate dehydrogenase enzymatic method. Circles represent the values of individual mice, diamonds represent means, and bars are SEM. N = 9, 8, 11 and 10 for vehicle (Control), ezetimibe (EZE), atorvastatin (ATO), and combination (EZE+ATO) groups, respectively. Statistical analysis was performed using one way repeated measure ANOVA. ** P<0.05; compared with vehicle. (TIF) [file pone.0142430.s004.TIF]

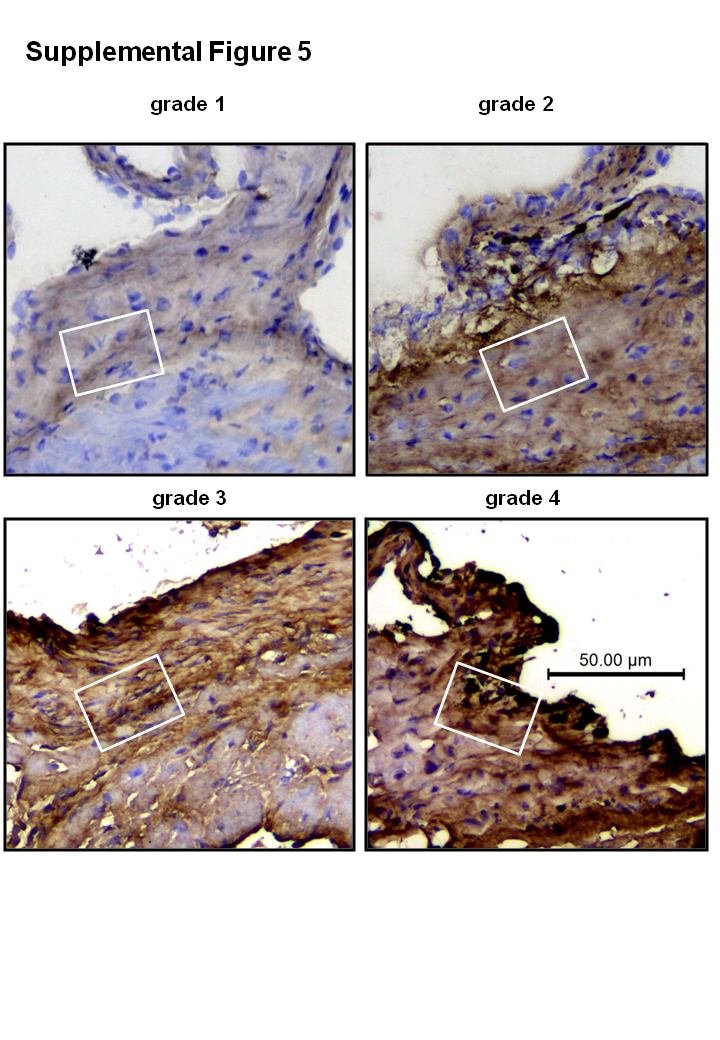

Supplement: S5 Fig — In the lesions, macrophage (CD68) was detected by immunostaining on cross-sections (10 μm thick) of aortic roots. Macrophage content in atherosclerotic lesions was graded as follows: 0 = no staining, 1 = slight staining, 2 = mild staining, 3 = moderate staining and 4 = abundant staining. Artificial classification (grade 1–4) by immunostaining was used to evaluate macrophage contents in the lesions. CD68 positive staining as shown brown in the lesions represents macrophage. (TIF) [file pone.0142430.s005.TIF]

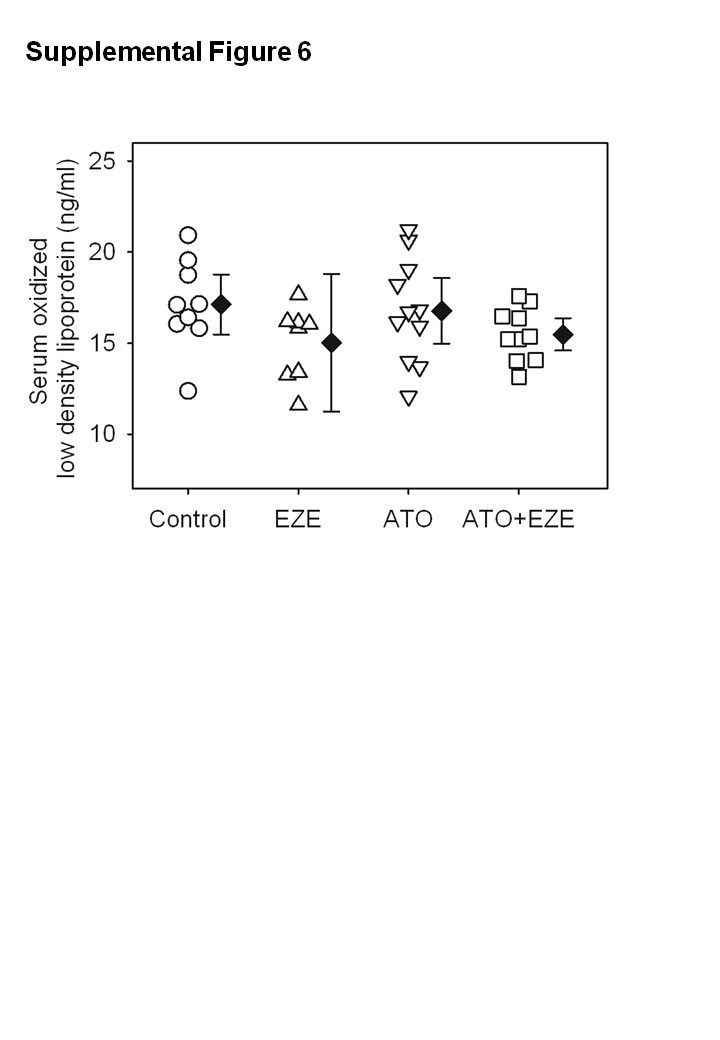

Supplement: S6 Fig — Serum oxLDL concentrations were measured using an ELISA kit. Circles represent the values of individual mice, diamonds represent means, and bars are SEM. N = 9, 8, 11 and 10 for vehicle (Control), ezetimibe (EZE), atorvastatin (ATO), and combination (EZE+ATO) groups, respectively. Statistical analysis was performed using one way repeated measure ANOVA. (TIF) [file pone.0142430.s006.TIF]
